# Transplant eligibility for patients with affective and psychotic disorders: a review of practices and a call for justice

**DOI:** 10.1186/s12910-017-0235-4

**Published:** 2017-12-08

**Authors:** Katherine L. Cahn-Fuller, Brendan Parent

**Affiliations:** 1 NYU Langone Health, Division of Medical Ethics, New York, NY USA; 2 Columbia University Medical Center, New York, NY USA

**Keywords:** Organ transplantation, Psychiatric illness, Psychosocial evaluation, Transplant candidates, Transplant eligibility

## Abstract

**Background:**

The scarcity of human organs requires the transplant community to make difficult allocation decisions. This process begins at individual medical centers, where transplant teams decide which patients to place on the transplant waiting list. Each transplant center utilizes its own listing criteria to determine if a patient is eligible for transplantation. These criteria have historically considered preexisting affective and psychotic disorders to be relative or absolute contraindications to transplantation. While attitudes within the field appear to be moving away from this practice, there is no data to confirm that eligibility criteria have changed.

**Main body:**

There are no nationwide guidelines detailing the manner in which affective and psychotic disorders should impact transplant eligibility. Individual transplant centers thus form their own transplant eligibility criteria, resulting in significant inter-institution variability. Data from the 1990s indicates that the majority of transplant programs considered certain psychiatric illnesses, such as active schizophrenia, to be absolute contraindications to transplantation. A review of literature reveals that no comprehensive data has been collected on the topic since that time. Furthermore, the limited data available about current practices suggests that psychiatric illness continues to be viewed as a contraindication to transplantation at some transplant centers. In light of this finding, we review psychiatric literature that examines the impact of affective and psychotic disorders on transplant outcomes and conclude that the presence of these disorders is not an accurate predictor of transplant success. We then discuss the requirements of justice as they relate to the creation of a just organ allocation system.

**Conclusion:**

We conclude that transplant eligibility criteria that exclude patients with affective and psychotic disorders on the basis of their psychiatric diagnosis alone are unjust. Just listing criteria must incorporate only those factors that have a causative effect on posttransplant morbidity and mortality. Justice also demands that we eliminate current inter-institution practice variations in favor of national transplant eligibility criteria. Given the limited data available about current practices, we call for an updated study investigating the manner in which affect and psychotic disorders impact transplant eligibility determinations.

## Background

There are over 120,000 people listed on the national transplant waiting list in the United States today. Less than one-third of these patients will receive a transplant by year-end and 22 people die on average each day [1]. As demand for transplantation grows, the gap between organ supply and demand widens and patients face longer waiting periods. The scarcity of human organs presents many ethical dilemmas and requires the transplant community to make difficult allocation decisions. This process begins at individual medical centers, where transplant teams decide which patients to place on the transplant waiting list.

Each transplant center utilizes its own listing criteria to determine if a patient is eligible for transplantation. These criteria have historically considered preexisting affective and psychotic disorders to be relative or absolute contraindications to transplantation [2]. While attitudes within the field appear to be moving away from this practice in favor of a more flexible approach to patient evaluation, there is no data to confirm that eligibility criteria have indeed changed. An Ovid MEDLINE search to January 2017 of the following terms revealed that there has been no comprehensive study of the patient selection criteria employed by US-based transplant programs for over 20 years: [transplantation OR organ transplantation OR transplantation, homologous] AND [mental disorders OR mental health OR anxiety disorders OR schizophrenia OR depressive disorder OR bipolar disorder OR depression OR psychotic disorder OR mood disorder] AND [eligibility determination OR eligible OR eligibility OR psychosocial OR patient selection OR waiting lists]. Furthermore, the limited data available about current policies indicates that liver transplant providers do not share consensus on the degree to which psychiatric illness should be viewed as a contraindication to transplantation [3].

This paper argues that the practice of barring patients with affective and psychotic disorders from transplantation is unjust and calls for an updated investigation into current patient selection criteria. We do not discuss the relationship between transplant eligibility and other psychiatric disorders, such as personality disorders, addiction, and intellectual disability. The paper begins with an overview of the available information regarding how affective and psychotic disorders are incorporated into eligibility criteria at the national and institutional level. We then review psychiatric literature that examines the effects of affective and psychotic disorders on transplant outcomes and determine that these studies do not support the exclusion of mentally ill patients from transplant waiting lists. Next, we discuss the requirements of justice and argue that policies that preclude patients with affective and psychotic disorders from transplantation are discriminatory. Finally, we call for the adoption of just and uniform eligibility criteria and highlight the need for updated data about current practices.

## Main text

### Transplant eligibility of patients with psychiatric illness

In 1984, the U.S. Congress passed the National Organ Transplant Act to create a national system by which donor organs are matched with patients waiting for transplants across the country. This act established the Organ Procurement and Transplant Network (OPTN) to maintain a national registry that facilitates the organ procurement and allocation process. The OPTN aims to provide equitable access to transplants, increase the number of transplants, improve patient outcomes, and promote efficient management of the transplant network. Since 1986, the United Network for Organ Sharing (UNOS), a private nonprofit organization, has contracted with the federal government to administer the OPTN [4].

All patients with organ failure who wish to receive an organ from a deceased donor or through kidney paired donation must register on the OPTN national patient waiting list. Once on the list, a patient is eligible to receive organ offers when suitable donor organs become available [5]. In order for a patient to gain access to the waiting list, a multidisciplinary transplant team must evaluate the patient to determine if she is eligible for transplantation. Eligibility criteria include both medical and psychosocial factors.

The impact of a patient’s history of affective and psychotic disorders on her transplant eligibility differs amongst transplant centers. While UNOS requires all transplant programs to identify appropriately trained individuals to perform psychosocial evaluation of potential transplant recipients, the organization does not specify the content of such evaluations [6]. The official policies of the OPTN offer even less guidance, and provide no discussion of the need for psychosocial evaluation [5]. Despite this absence, the U.S. Department of Health and Human Services’ Centers for Medicare & Medicaid Services (CMS) recognizes the impact that psychosocial factors have on the prognosis of organ transplant recipients. Accordingly, the CMS requires transplant centers to provide every transplant candidate with a comprehensive psychosocial evaluation prior to placement on the waitlist. A transplant center must meet this requirement to be eligible for reimbursement for transplant services [7].

To help transplant centers interpret this requirement, professional associations offer their own guidelines (Table 1). The Practice Guidelines released by the American Association for the Study of Liver Diseases (AASLD) recommend that all patients undergo psychosocial evaluation to ensure that they meet reasonable expectations for adherence to medical directives and mental health stability. While the guidelines recognize that psychiatric disorders may be associated with poor outcomes, the AASLD states: “There is no psychiatric disorder that is an absolute contraindication to transplantation and even the most psychiatrically complex patient… can have successful long-term outcomes” [8]. The International Society for Heart and Lung Transplantation’s (ISHLT) listing criteria for heart transplantation recommend that psychosocial evaluations assess a patient’s ability to give informed consent and comply with medical instruction. The Society views demonstrated medical noncompliance as an absolute contraindication to transplantation, and states that poor social support is a risk factor for noncompliance and is thus a relative contraindication to transplantation: “Any patient for whom social supports are deemed insufficient to achieve compliant care in the outpatient setting may be regarded as having a relative contraindication to transplant” [9]. Similarly, the ISHLT guidelines for the selection of lung transplant candidates lists “psychiatric or psychologic conditions associated with the inability to cooperate with the medical/allied health care team and/or adhere with complex medical therapy” as an absolute contraindication to transplantation [10]. Finally, the American Society of Transplantation (AST) guidelines for the evaluation of renal transplant candidates recommend an experienced evaluator screen each patient for a personal and family history of mental illness. The Society states that mental illness is not an absolute contraindication to transplantation, and “caregivers should attempt to reduce psychiatric barriers to successful transplantation” through appropriate diagnosis, treatment, and follow-up care [11]. The reason for these discrepancies is unclear, and likely relates to the success rate and complications of each type of transplantation. A thorough examination of the development of these guidelines may prove to be an important direction for future research.

**Table 1:** Professional guidelines for the inclusion of psychosocial factors in transplant eligibility criteria

Association	Organ	Contraindications
American Association for the Study of Liver Diseases (2013)	Liver	Absolute contraindications: • None Relative contraindications: • Psychiatric illness: “There is no psychiatric disorder that is an absolute contraindication to transplantation” [8]. • Medical noncompliance: “Patients should be evaluated for and meet reasonable expectations for adherence to medical directives and mental health stability” [8]. • Poor social support: “Patients should have adequate social/caregiver support to provides the necessary assistance both while waitlisted and until independently functioning in the postoperative period” [8].
International Society for Heart and Lung Transplantation (2016)	Heart	Absolute contraindications: • Medical noncompliance: “Patients who have demonstrated an inability to comply with drug therapy on multiple occasions should not receive transplantation” [9]. Relative contraindications: • Poor social support: “Any patient for whom social supports are deemed insufficient to achieve compliant care in the outpatient setting may be regarded as having a relative contraindication to transplant” [9].
International Society for Heart and Lung Transplantation (2014)	Lung	Absolute contraindications: • Medical noncompliance: “Psychiatric conditions associated with the inability to cooperate with the medical/allied health care team and/or adhere with complex medical therapy” [10]. • Poor social support: “Absence of an adequate or reliable social support system” [10]. Relative contraindications: • None
American Society of Transplantation (2001)	Kidney	Absolute contraindications: • None Relative contraindications: • Psychiatric illness: “Renal transplant candidates with a history of mental illness should undergo evaluation, counseling and, if necessary, treatment by appropriate mental health professionals prior to transplantation” [11]. • Medical noncompliance: “It is reasonable to delay transplantation for patients who, despite interventions, are not able to improve life-threatening, noncompliant behaviors” [11].

While these professional societies offer more detailed descriptions of psychosocial evaluations than does the OPTN, their guidelines are neither mandatory nor comprehensive. As a result, individual transplant centers are left to form their own criteria for the evaluation of transplant candidates. Given this autonomy, transplant programs exhibit a great deal of variation in the methods used for patient assessment and the content of transplant eligibility criteria [12]. The majority of these programs use clinical interviews, conducted by trained nurses, physicians, psychologists, or social workers, to complete the assessment. Kidney transplant programs are less likely to employ psychiatrists and psychologists than are cardiac and hepatic programs, and often rely on social workers [2]. Interviewers sometimes use rating scales, such as the Psychosocial Assessment of Candidates for Transplant (PACT) and the Transplant Evaluation Rating Scale (TERS), to supplement the clinical interview. Both PACT and TERS use information gained during psychosocial assessment to determine a patient’s suitability for transplantation [12]. Few centers use formal psychological testing during the screening process, although this practice is more common in cardiac transplantation programs than in liver and kidney transplantation programs [2].

Limited information is available about the specific selection criteria used by transplant centers across the nation. The most recent comprehensive data comes from Levenson and Olbrisch, who published two studies in the 1990s comparing the psychosocial evaluations used by cardiac, liver, and kidney transplant programs [13, 14]. According to their research, cardiac programs had the most stringent criteria for listing candidates on the organ waiting list, followed by liver and then kidney programs. The relatively accommodating criteria of renal programs may be explained by the long history of renal transplantation and the tendency of transplant programs to adopt more lenient selection criteria over time. More than 50% of cardiac programs recognized a number of psychosocial conditions as an absolute contraindication to transplantation, including: active schizophrenia (defined as schizophrenia with active psychotic symptoms), a recent suicide attempt, a history of multiple suicide attempts, current suicidal ideation, and medical noncompliance. Both active schizophrenia and current suicidal ideation were also listed as absolute contraindications for at least half of renal and liver transplant programs. Common relative contraindications to transplantation included: a family history of mental illness, controlled schizophrenia, current affective disorder, a history of affective disorder, a recent suicide attempt, history of one or more suicide attempts, poor social support, and medical noncompliance. Overall refusal rates following psychosocial evaluation ranged from 0 to 40% across programs, averaging 5.6%, 2.8%, and 3.0% for cardiac, livers, and renal programs, respectively. Of note, a program’s refusal rate did not correlate with its list of psychosocial contraindications and may be better explained by variations in the evaluation process, available psychosocial resources, and patient population at each center. Levenson and Olbrisch also collected data from non-US cardiac transplant programs, which were more likely to deem patients with controlled schizophrenia and medical noncompliance ineligible for transplantation. However, international programs were more lenient about other criteria, such as poor social support, and were less likely than US cardiac programs to refuse a patient for transplantation on psychosocial grounds [2].

While the data published by Levenson and Olbrisch is now more than 20 years old, there are no newer studies that provide a comparable, comprehensive look into the practices of transplant programs across the nation. The most relevant modern publication comes from Secunda et al., who assessed the opinions of US transplant providers regarding the way in which psychosocial characteristics influence patient eligibility for liver transplantation [3]. The authors found that psychiatric diagnoses were one of the most controversial characteristics impacting a patient’s eligibility for transplantation, with approximately half of survey respondents marking the characteristic as controversial. Furthermore, transplant providers demonstrated significant disagreement when asked to identify psychiatric concerns as absolute or relative contraindications to transplantation. For example, 110 survey respondents marked unstable psychiatric illness as an absolute contraindication while 128 respondents considered this characteristic to be a relative contraindication to transplantation. Significant disagreement was likewise seen when respondents were asked about stable psychiatric illness and a prior suicide attempt, with respondents divided as to whether such characteristics were either not a contraindication or a relative contraindication to transplantation. Secunda and colleagues also found that less than half of respondents indicated that their transplant center had a written policy about how psychiatric diagnoses should impact a patient’s transplant eligibility. Although this study focused exclusively on liver transplantation, it indicates that some of the exclusionary practices found by Levenson and Olbrisch may still be in place today. The variability of respondents’ opinions also highlights the presence of inter-institution inconsistency, creating disparity in the treatment of similarly situated individuals across transplant centers, and raises questions about the extent to which current practices follow the recommendations of professional associations.

### Impact of psychiatric illness on transplant success

In light of the data indicating that a history of psychiatric illness may impact a patient’s placement on the transplant waiting list, it is important to investigate the impact of affective and psychotic disorders on transplant outcomes. Because pretransplant psychosocial screenings seek to evaluate patients’ readiness for transplantation, the inclusion of psychiatric characteristics in transplant eligibility criteria illustrates the belief that certain psychiatric patients experience increased posttransplant morbidity and mortality. Psychiatric illness is thought to negatively impact transplant outcomes through a number of mechanisms, including: poor adherence to medication regimes, interpersonal difficulties that lead to poor social support, self-injurious behaviors, and drug-drug interactions between psychiatric and immunosuppressant medications [15]. Given the limited supply of organs, these concerns may cause some transplant centers to exclude certain psychiatric patients from transplantation in favor of patients who are more likely to be successful recipients and responsible stewards of their new organs. However, data about posttransplant outcomes of patients with affective and psychotic disorders illustrate that these psychiatric illnesses are not consistently associated with increased morbidity and mortality. In what follows, we examine peer-reviewed studies that both support and refute the association between psychiatric illness and poor transplant outcomes. We first discuss this relationship with reference to affective disorders and then turn to psychotic disorders.

A number of recent studies suggest that affective disorders negatively impact the survival of transplant recipients. Owen et al. evaluated the relationship between pretransplant psychiatric illness and posttransplant morbidity and mortality in patients undergoing cardiac transplantation [16]. The authors found that a shortened survival time was associated with current depressive disorder, a history of suicide attempts, and a history of poor medical adherence. A history of suicide attempts was also strongly associated with decreased time to infection and organ rejection. Overall, current depression was one of the strongest predictors of reduced posttransplant survival, conferring a threefold increase in mortality. DiMartini and colleagues found a similar pattern amongst liver transplant recipients [17]. Their prospective study followed patients transplanted for alcoholic liver disease, and found that those with a history of depression were at increased risk of depression after transplantation. Early posttransplant depression subsequently served as the strongest predictor of long-term patient survival. A recent review article further supports the association between pretransplant depression and increased risk of posttransplant mortality in various solid organ transplants [18].

There is also a body of evidence that contradicts these findings. Rosenberger et al. conducted a review examining the effect of pretransplant psychiatric disorders on transplant recipient morbidity and mortality [19]. The authors found that pretransplant depression did not negatively impact posttransplant survival in liver, lung, and kidney recipients. The effect of psychiatric illness on heart recipients was less clear, and suggested that patient mortality was affected by the interaction between depressive symptoms and heart failure etiology. Kidney recipients with pretransplant depression experienced an increased risk of return to dialysis. Psychiatric illness was not associated with increased morbidity in liver and heart recipients. Another recent study investigating the effect of depression in lung transplant recipients found that this patient population was not at increased risk of posttransplant complications such as bronchiolitis obliterans syndrome and graft loss [20].

Further evidence that psychiatric illness is not associated with adverse outcomes comes from Evan et al., who analyzed the morbidity and mortality of transplant recipients at the Veterans Health Administration (VHA) [21]. The study found that severe mental illness, including psychotic disorders, bipolar disorder, major depressive disorder, and severe posttraumatic stress disorder, did not impact posttransplant recovery. Veterans with pretransplant mental illness experienced the same rates of graft rejection and 3-year survival as recipients without mental health disorders. Mental illness also did not impact medical compliance, measured by attendance at postoperative outpatient appointments and by the number of immunosuppressive medication refills. The posttransplant mental health services and social supports available to transplant recipients at the VHA may contribute to these promising results. This finding reinforced that of an earlier study, which found that pretransplant depression was not a predictor of noncompliance in solid organ transplant recipients [22].

Few studies examine the outcomes of patients with psychotic disorders after transplantation. The relatively low lifetime prevalence of psychotic disorders, which is approximately 3%, and the limited transplants performed on psychotic patients make longitudinal studies of this patient population difficult. One report comes from Coffman and Crone, who surveyed transplant programs throughout the United States, Canada and Australia to collect data about transplant recipients with pretransplant psychotic disorders [23]. The survey yielded 35 cases at 12 transplant programs and included patients with schizophrenia, schizoaffective disorder, bipolar disorder, major depression with psychotic features, and psychotic disorder not otherwise specified. The authors found 13 of the 35 patients suffered from mania or psychotic episodes after transplantation, seven patients attempted suicide, and two patients completed suicide. Both suicide attempts and completion were more common in patients who experienced psychotic symptoms during the year prior to transplantation. Approximately one quarter of patients exhibited medication noncompliance after surgery, resulting in rejection episodes in five patients and in reduced function or graft loss in four patients. Of note, noncompliance with immunosuppressant drugs was highly correlated with living alone, homelessness, and time since last psychotic episode.

More recent studies suggest that patients with psychotic disorders may be successful transplant recipients. Zimbrean and Emre conducted a retrospective review that examined the impact of preexisting psychotic disorders on transplant outcomes [15]. They identified ten patients with a history of psychosis who received solid organ transplants and found that all patients were adherent with medication regimes and outpatient appointments following transplantation. Four patients experienced one episode of organ rejection each, none of which were associated with an exacerbation of psychotic symptoms, medication noncompliance, or graft loss. Psychiatric complications after transplantation included psychotic episodes, depression, mania, and substance abuse. Four patients required one or more psychiatric hospitalizations, with a mean number of 0.42 per patient per year of follow-up. No deaths occurred among the ten transplant recipients. Overall, the group showed no evidence of adverse medical events related to an exacerbation of psychotic symptoms. The authors hypothesized that the good outcomes seen in this study were influenced by the extensive psychiatric care offered to patients in the pretransplant and posttransplant settings. A number of case studies also demonstrate that patients with psychotic disorders may undergo successful transplantation if they receive the appropriate psychiatric and social support. Le Melle and Entelis describe a heart transplant recipient with active schizophrenia who was compliant with immunosuppresant medications and follow-up appointments after transplantation [24]. The patient did not suffer from any significant medical or psychiatric complications. DiMartini and Twillman report a similar case in which a patient with schizophrenia exhibited medical compliance and psychiatric stability following liver transplantation [25].

These results are difficult to compare given differences in methodology, study populations, and transplant selection criteria. For example, studies in which psychiatric patients experienced better posttransplant outcomes were more likely to provide extensive psychiatric care and employ targeted listing criteria. Nevertheless, it is clear that there is no consensus on the effect of affective and psychotic disorders on transplant outcomes. The relationship between psychiatric illness and specific risk factors, such as medical noncompliance, is likewise ambiguous. Psychiatric patients are an extremely heterogeneous group, and a patient’s psychiatric diagnosis alone is unlikely to be an accurate predictor of transplant success.

### The demands of justice

These conflicting studies illustrate that current evidence does not support the practice of barring patients with affective and psychotic disorders from transplantation on the basis of their psychiatric illness alone. While affective and psychotic disorders may be associated with poor transplant outcomes, they do not appear causative. Rather, poor posttransplant outcomes are likely caused by concomitant factors, such as medication noncompliance and insufficient social support. In this section, we discuss the demands of justice and examine how justice informs transplant eligibility criteria. We argue that selection criteria that exclude psychiatric patients are unjust and provide recommendations for the creation of a just organ allocation system.

There is currently no consensus on what constitutes the just distribution of scarce healthcare resources, and various theories of justice offer unique approaches to this dilemma. The OPTN/UNOS Ethics Committee defines justice as “the fair pattern of distribution of benefits” of an organ allocation program and suggests that organ allocation decisions must incorporate concerns of justice and utility [26]. The American Medical Association’s Council on Ethical and Judicial Affairs (CEJA) also emphasizes the importance of justice and utility and offers guidelines for organ allocation that reflect these concerns [27]. In line with these groups, we define a just allocation system as one that balances concerns of fairness and utility. Unlike the OPTN/UNOS Ethics Committee and CEJA, both of which use the terms justice and fairness interchangeably, we separate the two concepts and posit fairness as a necessary, but insufficient, component of just organ allocation.

Fairness demands that all potential transplant candidates are given full and equal consideration for placement on the organ waiting list according to a uniform set of eligibility criteria. This requires that all patients are evaluated according to their individual characteristics and medical history and are not dismissed on the basis of impersonal factors or membership to a social group. Of course, human organs are extremely limited and the principle of fairness alone is insufficient to guide organ allocation. As a result, we must balance considerations of fairness with those of utility. The principle of utility holds that organs should be allocated in a manner that maximizes the good produced. When forced to allocate an organ to one of many needy patients, utility tells us to select the recipient who will gain the most benefit from transplantation.

When considered in tandem, the principles of fairness and utility provide useful guidelines for the creation of a just organ allocation system. Utility restricts the subset of patients who are eligible for transplantation to those who are expected to gain a certain amount of benefit from the procedure. Here, benefit is best defined as increased life expectancy and improved quality of life. This restriction is necessary to ensure that organs are allocated in a manner that reflects the limited nature of the resource. Fairness, on the other hand, limits the extent to which concerns of utility guide allocation decisions. For example, we may accept that all transplant candidates must be expected to gain a specific minimum benefit from transplantation (e.g. ten additional years of life), but insist that considerations beyond this threshold are either less significant or morally irrelevant. Similarly, only substantial differences in patient outcomes should affect allocation decisions. Fairness also requires that transplant eligibility criteria incorporate only those factors that have a known impact on transplant success and avoid subjective judgments of a patient’s social worth.

In light of this discussion, it is evident that the exclusion of patients with affective and psychotic disorders from transplantation is unjust. This practice stems from the assumption that certain psychiatric diagnoses are associated with a set of characteristic traits and behaviors that will lead to worse posttransplant outcomes, thereby ignoring the heterogeneity of psychiatric patients. For example, it is often presumed that patients with psychotic disorders will be unable to adhere to posttransplant medication regimes and outpatient appointments. Given the strong association between medical noncompliance and increased morbidity and mortality, this presumption may lead to the creation of eligibility criteria that bar patients with psychotic disorders from gaining access to the transplant waiting list [12, 23]. Yet psychotic disorders themselves do not directly predict transplant success. Rather, it is the specific behavior of medication noncompliance that is relevant. In fact, studies demonstrate that the overall noncompliance rate of psychiatric patients falls within the range of noncompliance seen in the larger transplant population [23]. It is therefore unjust to label affective and psychotic disorders, the pathophysiologies of which do not directly compromise transplanted organs, as contraindications to transplantation.

This in no way implies that medical adherence is irrelevant to transplant candidacy. Rather, transplant teams should strive to assess a patient’s likelihood of noncompliance directly rather than assuming that psychiatric patients are necessarily at risk. This may be achieved through the evaluation of a patient’s pretransplant medical adherence and specific social support networks, both of which have been shown to be independent predictors of posttransplant noncompliance [28]. Future research on the predictors of medical adherence should likewise strive to identify risk factors that are proximal determinants of noncompliance. In turning our focus from broad categories of psychiatric illness to patient-specific risk factors, we can better predict which patients are likely to experience poor outcomes as well as avoid the unjust discrimination of psychiatric patients as a whole.

In addition to creating listing criteria that give weight to a patient’s individual behaviors and characteristics rather than his psychiatric diagnosis, justice also demands that we apply the same criteria to all patients equally. The current inter-institution variation in practices results in inconsistent eligibility decisions across transplant centers, thereby favoring patients who have the means to seek treatment at more than one hospital. To ensure that all potential transplant recipients are given equal consideration, a national transplant eligibility criteria and method of psychosocial assessment must be established. While such criteria should allow for some institutional variation based on factors such as a transplant program’s recent successes/failures and the availability of alternative treatments, a formalized standard is needed to ensure just organ distribution.

Finally, it is important to consider what is owed to psychiatric patients as a community that contributes to organ donation. Current regulations allow individuals with affective and psychotic disorders to serve as organ donors for both living and deceased organ donation without qualification on the grounds of their psychiatric conditions [29, 30]. While the role of reciprocity in organ donation is the subject of current debate, our willingness to accept organs from mentally ill donors surely imparts a moral obligation on the medical community to ensure that this patient population receives fair consideration when in need of organ transplants. Such consideration entails that transplant teams view the risky behaviors associated with psychiatric illness as potentially modifiable risk factors and seek to improve a patient’s suitability for transplant through the use of psychiatric and social resources. Recent studies support this approach to mental illness and demonstrate that improved psychiatric care reduces the incidence of posttransplant morbidity and mortality [15, 31]. Transplant teams that have the resources to address patients’ barriers to care should help patients meet eligibility criteria whenever possible.

### Objection

In response to our description of just organ allocation, one may argue that we are downplaying the importance of utility. Given the scarcity of organs, why risk allocating precious resources to psychiatric patients who may be at increased risk for poor outcomes? Furthermore, why waste organs on mentally ill patients who enjoy a lower quality of life than other transplant candidates?

We agree that concerns of utility are essential to allocation decisions, but believe that this objection is misguided. According to our suggestion, a psychiatric patient that demonstrates high-risk behaviors and is not expected to gain a minimum benefit from transplantation should not be considered a transplant candidate. This may be the case for a patient that is at high risk of completing suicide after transplantation or has a long history of medical noncompliance. However, medical professionals should not determine that a patient is ineligible for transplantation based on her psychiatric diagnosis alone. It is helpful to compare our treatment of patients with affective and psychotic disorders to that of adolescents. Like psychiatric patients, adolescents are at risk of noncompliance with immunosuppressive medications. A number of studies demonstrate that the prevalence of noncompliance is higher in adolescents than both adult and pediatric transplant recipients. The cause of noncompliance in this patient population is likely multifactorial and includes factors such as age-related risk-taking behavior and a desire to avoid the cosmetic side effects of immunosuppressive medications [32]. We do not, however, consider adolescence to be a contraindication to transplantation. Rather, medical providers determine if young patients are eligible for transplantation on an individual basis and recognize the importance of behavioral and psychosocial interventions aimed at enhancing adherence. While such measures may not ensure compliance, they lessen the risk of noncompliance to a level that the transplant community is willing to tolerate. We ask that institutions treat psychiatric patients in a similar manner, evaluating each as an individual and offering supportive services to curb risky behavior. In so doing, the transplant community will be able to balance concerns of utility with fairness and avoid systematically marginalizing members of our society.

It is likewise inappropriate to discount transplant candidates with psychiatric illness based on the assumption that mentally ill patients necessarily enjoy a low quality of life, or live a life that is less valuable than those of other members of society. Quality of life considerations are certainly relevant to transplant eligibility, and just listing criteria may require all transplant candidates to gain an expected minimum net improvement in transplant-related quality of life to be eligible for transplantation. However, quality of life and value of life measures are inherently subjective, and it is not the role of medical professionals to determine which patients’ lives are worth living. For example, consider a patient with a history of depression suffering from end-stage renal disease. If a kidney becomes available but it is deemed that transplantation will not enable the patient to discontinue dialysis, the medical team may justly determine that the patient will not gain a sufficient quality of life improvement from the transplantation. In this case, it is clear that the new kidney will not add significant biological or functional value to the patient’s life. If we modify the example such that the transplant will enable the patient to come off dialysis but will not prevent her from experiencing depressive episodes throughout her lifetime, the transplant team should not deem the patient ineligible for transplant based on the fact that the procedure will not prevent future depressive episodes. Doing so would unfairly substitute subjective quality and value of life judgments for objective quality of life judgments. There are no objective criteria with which to measure the value of an individual’s lived experience. Any attempt to create such criteria would fail, as no individual is capable of experiencing another’s life. As a result, subjective quality of life and value of life considerations that lie outside the scope of transplant-related outcomes must not influence listing criteria.

## Conclusions

Transplant eligibility criteria that exclude patients with affective and psychotic disorders from transplantation on the basis on their psychiatric diagnosis alone are unjust. This practice penalizes patients for their association with a diagnostic category and discourages transplant providers from considering patient-specific behaviors and characteristics that contribute to transplant outcomes. We argue that just listing criteria must incorporate only those factors that have been demonstrated to have a causative effect on posttransplant morbidity and mortality. Justice also demands that transplant centers evaluate all potential candidates with a standard set of listing criteria and offer medical and social support to patients who fail to meet these criteria in an effort to overcome obstacles to treatment.

We suspect that many professionals involved in organ allocation decisions will find our conclusion to be uncontroversial. In fact, our survey of peer-reviewed studies reveals that many transplant centers already transplant patients with a history of psychiatric illness. The practice of performing liver transplants on patients who require the procedure because of an intentional acetaminophen overdose likewise illustrates the willingness of some medical professionals to transplant psychiatrically ill patients [33]. However, the current attitudes and practices of those involved in transplant eligibility determinations are unknown, as there are no recently published studies on the subject. As already discussed, the available nationwide data is now more than 20 years old and revealed significant heterogeneity in the treatment of patients with psychiatric illness at that time [13, 14]. The limited data available on the attitudes of medical professionals today demonstrates that psychiatric diagnoses remain some of the most controversial characteristics impacting a patient’s eligibility for transplantation [3]. Given the uncertainty surrounding current practices, we aim to provide a comprehensive, empirically informed ethical argument in support of the inclusion of patients with affective and psychotic disorders on transplant waiting lists. We also call for an updated study investigating the manner in which affective and psychotic disorders impact eligibility determinations as they are made today. Only when this information is available can the transplant community judge its practices according to the demands of justice. It is our hope that the ethical concerns presented in this paper will serve as a lens through which to critique eligibility determinations as well as a guide for future practice guidelines.

## Abbreviations

AASLD: American Association for the Study of Liver Diseases
AST: American Society of Transplantation
CEJA: Council on Ethical and Judicial Affairs
CMS: Centers for Medicare & Medicaid Services
ISHLT: International Society for Heart and Lung Transplantation
OPTN: Organ Procurement and Transplant Network
PACT: Psychosocial Assessment of Candidates for Transplant
TERS: Transplant Evaluation Rating Scale
UNOS: United Network for Organ Sharing
VHA: Veterans Health Administration
